# The Future of Genetic Disease Studies: Assembling an Updated Multidisciplinary Toolbox

**DOI:** 10.3389/fcell.2022.886448

**Published:** 2022-04-28

**Authors:** Swetha Ramadesikan, Jennifer Lee, Ruben Claudio Aguilar

**Affiliations:** Department of Biological Sciences, Purdue University, West Lafayette, IN, United States

**Keywords:** genetic diseases, vesicle trafficking, protein sorting, cell biology, gene mutation

## Introduction and Summary

Over the past decades, we witnessed impressive progress in our understanding of the mechanisms behind multiple genetic diseases including many considered to be “rare.” Importantly, these advances coupled with new technological developments have increased the potential to translate research into tangible improvements in diagnosis and treatment. Here, we argue that to further accelerate discovery and translational applications in the field, the classical genetic, biochemical, and cell biological toolbox must be updated by systematic integration of rising methodologies from different disciplines. This short opinion article aims to encourage clinicians, geneticists, biochemists, and cell biologists to discover the impact and consider the methodical integration of each other’s state-of-the-art disciplines in single comprehensive studies. Indeed, studies systematically integrating such emergent powerful approaches (see below) are not frequent in the field yet, perhaps because such strategies require investment in expanding one’s expertise/repertoire of technologies (some of them seemingly very specialized) and in building communication bridges between disciplines perceived as distant (Love et al., 2021). Nevertheless, we consider that these are worthy efforts as the full use of an updated multidisciplinary methodological toolbox will assure future breakthrough studies. These diverse approaches, commonly used at the different steps of the scientific inquiry, are needed to formulate mechanistic hypotheses and consequently, to develop therapeutic strategies. Further, these methodologies are also expected to be used *a posteriori* to evaluate the efficacy of such therapeutic strategies. In fact, several studies using at least a subset of powerful and complementary approaches have already yielded important observations and conclusions, some of which are exemplified below.

## Candidate Components for an Updated Multidisciplinary Toolbox: Examples and Lessons Learned

Although the role of proteins in specific diseases has often been studied by production of gene knock-out (KO) cells or animals, many patients present mutated protein variants rather than complete absence of gene product. The application of NGS technologies such as whole genome/exome sequencing (WGS/WES) has been (Ng et al., 2009; Ng et al., 2010), and continues to be (Macken et al., 2021; Marom et al., 2021; Usmani et al., 2021) instrumental in the identification of novel mutations including those leading to splicing defects. Further, this mutational information can be efficiently analyzed using advanced algorithms to predict non-tolerated/pathogenic changes (Ng and Henikoff, 2003; Schwarz et al., 2010; Adzhubei et al., 2013; Ioannidis et al., 2016; Rentzsch et al., 2019; Ge et al., 2021) and by molecular dynamics to infer putative structural alterations (Kellogg et al., 2011; Adolf-Bryfogle and Dunbrack, 2013).

Nevertheless, to understand the mechanisms underlying such pathologies, we must establish the potential impact of specific gene mutations on the *function* (or regulation of function) and/or on the *availability* (from absence to altered dosage, but also by incorrect localization) of the resulting protein. Moreover, examples from literature indicate that mutations assumed to be similar and affecting the same protein domains can display phenotype heterogeneity (Arystarkhova et al., 2019; Arystarkhova et al., 2021; Ramadesikan et al., 2021). The necessity of firmly establishing a genotype-phenotype relationship is one reason why the articulation of genetic/biochemical methods with cell biological approaches in particular, are a must.

Indeed, the use of classical functional biochemical assays (Davies et al., 2013; Fausther et al., 2014; Guttmann et al., 2018; Saveri et al., 2020) has been successfully complemented with powerful functional cell biological assays. Further, the latter provides context and physiological relevance for the functional impact of mutations in different genes, can be used as a diagnostic tool and for the evaluation of therapeutics (Bergamin et al., 2013; Visentin et al., 2013; Kwiatkowska et al., 2014; Vanier and Latour, 2015; Aoki et al., 2017; Carrion et al., 2017; Saveri et al., 2020; Wanikawa et al., 2020; Hung et al., 2021; Ramadesikan et al., 2021; Romano et al., 2021).

Moreover, the cumulative work of several groups has provided clues about the suitability of different disease models. On one hand, due to adaptation and “dominant” negative effects, the presence of a mutated variant can cause different and/or more severe phenotypes than the absence of the protein (as modeled by KO) (Tucker et al., 2012; Barnes et al., 2018; Lizarraga et al., 2021; Ramadesikan et al., 2021). On the other hand, cell biologists have long known that phenotypes observed under siRNA-mediated knock-down conditions (*i.e.,* acute depletion over a short period of time) often lead to more severe phenotype scenarios due to lack of cell/organism adaptation. Therefore, this confirms that when appropriate, *complete lack of function scenarios* can be better modeled by gene KO or (in some cases) by stable expression of shRNAs rather than by use of siRNAs.

Further, the CRISPR revolution has provided the means to reproduce and study pathological protein variants within meaningful and physiologically relevant contexts (Freedman et al., 2015; Ponomareva et al., 2016) as well as perform targeted rescue of disease-causing mutations in model systems (Min et al., 2020). These developments become synergistic with the possibility of reprogramming patient-derived induced Pluripotent Stem Cells (iPSCs) to study cell biological behavior of mutated variants expressed at endogenous levels in disease-relevant cell types and organoids (Parfitt et al., 2016; Boutry et al., 2018; Dvela-Levitt et al., 2019; Flemming et al., 2020; De Rus Jacquet et al., 2021; Romano et al., 2021).

An important contribution of cell biology analyses to our understanding of the impact of missense and truncation mutations to pathological conditions relates to protein *availability*. For example, mislocalization and delayed traffic leads to lack of protein availability when and where the target is needed causing phenotypic manifestations (Jacoby et al., 2009; Braun and Schweizer, 2017; Dunmore et al., 2020; Ramadesikan et al., 2021). Therefore, steady state and time-lapse microscopy localization studies are required (Wiegerinck et al., 2014; Bono et al., 2020; Murakami et al., 2020; Rodger et al., 2020; Mamais et al., 2021). These methodologies also open the possibility to be used as readouts for high throughput screens to identify affected biochemical circuits and/or candidate therapeutic agents.

Although currently used in only a few studies, techniques such as superresolution (Sumya et al., 2021; Zimmer et al., 2022) and light-sheet (Adhya et al., 2021) microscopies are already showing high value for genetic disease research. In addition, other important technical approaches used in various disciplines such as intravital microscopy (Frattolin et al., 2021), cell tracking (Betjes et al., 2021), atomic force microscopy (Dufrêne and Pelling, 2013; Sharma et al., 2018), single molecule imaging (Liu et al., 2022) and cryoelectron microscopy (Douguet et al., 2019) or tomography (Shoemark, 2017) are expected to increase in utilization for mechanistic studies related to genetic diseases.

Moreover, the specific case of mutations affecting protein sorting signals (Mukherjee et al., 2012; Aguilar, 2015) is likely not to be detected by routine sequence analysis and standard biochemical approaches, requiring appropriate studies and interpretation of intracellular localization data to reveal cellular abnormalities. Along the same lines, evaluating the specific role of a mutated protein potentially involved in protein traffic/sorting in a disease (e.g., coatopathies (Dell’angelica and Bonifacino, 2019)) also requires cell biological analysis and expertise. Indeed, proficiency in this field and relevant approaches are needed to differentiate true sorting defects from other effects of mutations (such as ER-retention due to membrane-protein misfolding) that do not impact sorting signal recognition or the functionality of the trafficking machinery.

## Advances Towards the Use of an Integrated and Updated Multidisciplinary Toolbox

The previous section enumerates some approaches and novel developments involving the different steps required for constructing hypotheses about the genetic disease mechanism and framework for therapy design. Although integration of such methods in single multidisciplinary studies constitute an ideal scientific strategy to test overarching hypotheses, published examples more commonly involve using only some of the above nominated approaches. These partially integrated views are, of course, valuable and can be composed to yield an enhanced overview of the disease.

For example, a recent study identified mutations in *VPS4A* associated with a new multisystem disorder (with the proposed name CIMDAG: Cerebellar hypoplasia and cataracts, Intellectual disability, Microcephaly, Dystonia and Anemia, Growth retardation) showing striking neurodevelopmental abnormalities (Rodger et al., 2020). *VPS4A* encodes an ATPase that plays a vital role in ESCRT-III regulation and subsequent vesicle budding into multi-vesicular bodies. Novel *de novo* patient mutations were identified using WES/WGS approaches and their impact was analyzed using computational tools such as REVEL (Ioannidis et al., 2016) and CADD (Rentzsch et al., 2019). A thorough investigation of the pathogenesis mechanisms included assessing protein stability, localization in patient fibroblasts (Rodger et al., 2020). The authors demonstrated that while patient mutations did not impact Vps4a stability, they induced an increase in the fraction of enlarged endosomes (displaying different markers) and retention of the atypical ESCRT-III protein Ist1 (Increased Sodium Tolerance 1—involved in membrane fission) in the early endosomal compartment. Further, the study also characterized cellular phenotypes (including abnormalities in cell cycle and mitotic spindles, cilia and centrosome morphology) caused by a loss of Vps4a using CRISPRi in iPSC cell lines and neurons differentiated from these cell lines. These investigations provided clues into cellular and molecular mechanisms that may explain various clinical symptoms seen in patients. For example, numerous clinical features seen in ciliopathies have been observed in these patients and can be associated with defects in ciliogenesis while abnormalities in centrosome/mitotic spindle morphology may explain microencephaly observed in CIMDAG (Rodger et al., 2020).

Another example is the case of research applied to the study of Lowe syndrome (LS). This lethal genetic disease caused by abnormal function of the lipid phosphatase Ocrl1 (Oculo-Cerebro-Renal syndrome of Lowe) is characterized by mental retardation, bilateral congenital cataracts, and renal dysfunction (Recker et al., 2013).

The use of WES/WGS (Duran et al., 2016; Zheng et al., 2019) led to the discovery of novel LS causing mutations in the *OCRL1* gene, including several in non-coding regions, while molecular dynamics predicted structural consequences of such mutations (Ramadesikan et al., 2021). These studies were complemented by biochemical and cell biological assays to assess the mutational impact on protein function and availability (De Leo et al., 2016; Ramadesikan et al., 2021). Furthermore, models were prepared by various methods to gain mechanistic insight into LS such as KO/morpholino zebrafish lines (Coon et al., 2012; Ramirez et al., 2012; Gliozzi et al., 2020), humanized mouse models (Bothwell et al., 2011), Ocrl1-deficient cells by CRISPR (Madhivanan et al., 2020) along with stable lines expressing specific variants (Ramadesikan et al., 2021), and patient-derived iPSCs (Barnes et al., 2018; Hsieh et al., 2018; Liu et al., 2020; Akhtar et al., 2022). Taken together, LS research has greatly benefitted from the assimilation of a variety of techniques and is rapidly moving towards understanding LS in a patient mutation-specific manner to better guide the design novel therapeutic approaches.

## Final Remarks

In summary, we predict that the systematic utilization of an updated multidisciplinary toolbox including advanced approaches and multi-pronged strategies to study genetic disease will make possible the next quantum leaps in the understanding and treatment of genetic conditions.

Although we can accomplish these objectives by expanding/diversifying our own expertise, the more likely and efficient approach is to assemble multidisciplinary teams. For example, our (and others’) research in Lowe syndrome greatly benefitted from successful collaborations with bioinformaticians, computer scientists, structural biologists, nephrologists, and clinicians. Nevertheless, the success of this team strategy ultimately depends on one’s ability to face the challenge of providing proper and fair articulation of different disciplines and different scientific cultures (sometimes suffering serious communication issues and “cultural shocks”).

Carefully written Collaboration plans where strategies for communication, management, conflict prevention, self-assessment, etc., are stated (Hall et al., 2019), along with a clear shared view of the project can substantially contribute to cohesiveness of the team and facilitate the pursuit of complex scientific goals (Bennett and Gadlin, 2012; Love et al., 2021). Indeed, collaboration plans are already being required and evaluated by several funding agencies and are likely to become widespread mandatory in the immediate future. Here is where the findings of team science and its emerging strategies (Bennett and Gadlin, 2012; Love et al., 2021) can move our research beyond the sum of disciplines to enter the realm of synergism.
